# Burnout syndrome in health-care professionals in a university hospital

**DOI:** 10.6061/clinics/2017(05)08

**Published:** 2017-05

**Authors:** Lucila Corsino de Paiva, Ana Carla Gomes Canário, Eneluzia Lavynnya Corsino de Paiva China, Ana Katherine Gonçalves

**Affiliations:** I Universidade Federal do Rio Grande do Norte, Natal, RN, BR; II Faculdade Mauricio de Nassau, Natal, RN, BR

**Keywords:** Burnout Syndrome, Quality of Life, Occupational Health, Hospitals

## Abstract

**OBJECTIVE:**

To evaluate professional achievement and factors associated with occupational burnout among health professionals.

**METHODS:**

An institution-based cross-sectional study was conducted on 436 healthcare providers, consisting of 101 nurses, 81 doctors and 254 nursing technicians, all meeting pre-established inclusion criteria. Occupational burnout was detected using the Maslach occupational burnout inventory tool. Data were collected by self-administered questionnaires comprising questions concerning socio-demographics, education and training, and the Maslach occupational burnout inventory was used to identify levels of emotional exhaustion, depersonalization and professional achievement.

**RESULTS:**

Emotional exhaustion was associated with education level and work place for nursing technicians. Depersonalization was associated with gender in nursing technicians. For nurses, depersonalization showed a significant association with education level, whereas this factor was associated with number of jobs for doctors. Lower levels of professional achievement were observed for unspecialized doctors compared to those with further training. Higher levels of professional achievement were associated with professionals with postgraduate training compared to those without.

**CONCLUSIONS:**

High levels of emotional exhaustion were found in professionals from the maternity unit as well as in professionals with lower educational levels. Depersonalization was higher in physicians with several jobs and in female nurses. Low professional achievement was found in unspecialized doctors, while high professional achievement was associated with postgraduate training.

## INTRODUCTION

Burnout syndrome (BO) has long been recognized as a major problem within the professional sphere of modern life and has become much more prevalent in the last decade 1. This fact has generated enormous interest and concern, not only within the scientific community but also within government, business, and educational institutions due to the severity of the consequences that this syndrome has both on the individual and the work environment 1,2.

BO is a major psychosocial problem that affects professionals from different areas. It is caused by chronic stress in the work environment 2 and results in three distinct symptoms: emotional exhaustion (EE), depersonalization (DP) and reduced professional achievement (PA) 3.

EE, characterized by a lack of energy or feeling depleted and a lack of occupational motivation, is generally caused by personal conflict in relationships and a heavy workload. DP is a psychological state of emotional detachment wherein impersonal treatment of people in the workplace can give rise to a lackadaisical attitude, egocentric behavior, alienation, anxiety, irritability and demotivation. Reduced PA is characterized by a worker’s tendency to self-assess negatively, causing them to feel less competent and successful as a result and thus become dissatisfied with their PA 4.

Although BO affects individuals of all ages and occupations, it is highly prevalent among healthcare professionals due to the intense and continuous nature of contact with individuals receiving care 5. In addition, aspects such as age, gender, years of practice, interpersonal conflicts, training and low participation in decision-making have also been strongly associated with the syndrome 4,5. This is especially true of healthcare professionals working in hospitals, as their exposure to these occupational stressors is exacerbated by the nature of these institutions. The result is a negative impact on wellbeing in terms of both physical and mental health, which ultimately translates to substantial declines in several quality of life domains 6. Additionally, institutions suffer significant losses due to high levels of absenteeism from sickness 7 as well as presenteeism 8, which compromises the quality of service 9.

BO generally develops slowly, is triggered by multi-causal factors and is almost never identified in its early stages. Psychological changes such as difficulty in social relationships, moodiness, anxiety, and irritability are also frequent. Together, these factors can cause low productivity, conflicts in the workplace, dependence on psychotropic substances, and low PA. The end result is often high absenteeism or quitting, and BO can even lead to suicide in extreme cases if left untreated 10.

Although this syndrome has long been prevalent in healthcare professionals, studies have only recently started to evaluate cause and effect relationships as well as preventive measures 11. There is also no consensus in the existing literature on the prevalence and incidence of BO, although most studies have indicated a high prevalence in health professionals 5,12.

BO is still relatively misunderstood by the general population. However, it does merit attention due to the sheer number of people affected by it and the potential damage it can cause to individual and collective quality of life in the workplace 10,13. Considering its important implications regarding the physical and mental health of healthcare professionals, especially those working in hospitals, BO should be treated as a public health issue.

Additional studies are needed to better understand BO and to create a framework wherein professionals can qualify to treat and prevent the syndrome. Therefore, the present study assessed professional achievement and other factors associated with occupational BO among healthcare professionals.

## MATERIALS AND METHODS

This prospective study was conducted using convenience sampling in two hospitals that offer care, training and research in northeastern Brazil. The sample size was calculated based on 95% confidence intervals and with a margin of error not exceeding ±5 percent. Initially, 488 healthcare professionals were invited to answer a self-administered questionnaire; of these, 15 refused to participate and 37 did not meet the pre-established inclusion criteria. This resulted in a sample of 436 healthcare professionals, consisting of 101 nurses, 81 doctors and 254 nursing technicians.

The following inclusion criteria were established: working as a doctor, nurse or nursing practitioner and being in practice for more than five years at the institution. Those on vacation, sabbatical, sick leave, maternity leave, or away for professional training were excluded.

After discussing the study, the participants provided written informed consent and were given self-administered anonymous questionnaires to be completed during working hours according to their availability.

The questioning consisted of two parts: a questionnaire concerning socio-demographics, vocational training, and work followed by the Maslach BO inventory (MBI) 14, consisting of 22 questions concerning the different dimensions of BO that were translated to Portuguese and validated. This self-administered inventory is the most widely used and accepted instrument for assessing the key symptoms of BO.

We followed the criteria proposed by Ramirez et al. 15 and Grunfeld et al. 16 to verify the prevalence of BO and to obtain mean scores for EE, DP, and PA. The items on the BO instrument were answered on a five-point frequency scale ranging from 1 (never) to 5 (always). The questions evaluate 3 independent dimensions, including EE, DP, and PA. The sum of each dimension was obtained from the MBI scoring scale, and the results were classified according to values established by prior studies 13-17. EE scores ≥27 indicate extreme fatigue, those ranging form 19 to 26 correspond to moderate fatigue, and values <18 indicate a low level of fatigue. DP scores ≥10 were considered high, those ranging from 6 to 9 were considered moderate, and those <6 were considered low. PA scores ≥40 were considered high, those ranging from 34 to 39 were considered moderate, and those ≤33 were considered low 13-17.

A high level of BO corresponded to high scores for the EE and DP dimensions and low scores for the PA dimension. A moderate level of BO corresponded to average scores for all three dimensions. A low level of BO corresponded to low scores for the EE and DP dimensions and high scores for the PA dimension 18.

After collection, data were stored in a standardized format and digitalized for database management and statistical analysis, performed using SPSS version 20.0 for Windows.

Initially, an exploratory study was conducted to characterize the study population and identify factors related to the dimensions of EE, DP, and PA. Following this, the chi-square test and Fisher’s exact test for qualitative variables were applied to verify associations between sociodemographic, professional and work variables within the MBI dimensions. Odds ratios with 95% confidence intervals were used to measure the risk of developing BO.

This study was approved by the Ethics Committee of the Federal University of Rio Grande do Norte (UFRN), article 181 046, dated 21/12/2012 and CAAE 11674712.2.0000.5292.

## RESULTS

The majority of the sample was female (79.4%), over the age of 51 (33.0%), and married or in a stable relationship (62.8%). Most participants worked in the general hospital (73.8%), had a specialization (34.2%), and had been working there for 5 to 10 years (34.6%). The majority of professionals reported only one job (55.3%) and received an income greater than five minimum salaries (57.6%).

Three MBI dimensions were evaluated: EE, DP and PA. High EE was associated with low education level and was more common in nursing technicians from the maternity unit (*p*=0.04 and 0.01) (Table 1).

High DP was more common in female technicians (*p*=0.04) and those with higher education levels (*p*=0.02). In doctors, high DP was associated with a greater number of jobs (*p*=0.01) (Table 2).

Lower levels of PA were observed in unspecialized doctors compared to those with further training (*p*=0.03). Higher levels of PA were associated with postgraduate training (2.70; 95% CI: 1.40 to 5.20) compared to those without (1.74; 95% CI: 1.09 to 2.79) (Table 3).

## DISCUSSION

Although the work of healthcare professionals can be gratifying, factors such as work-life imbalance, long hours, perceived workload, distress caused by complaints and lack of reciprocity in relationships with patients and colleagues may lessen job satisfaction and consequently increase the risk of BO 19,20.

In this study, EE was associated with education level as well as work place for nursing technicians. EE was lowest in technical nurses working at the general hospital and highest in those working in the maternity unit. The maternity unit is a high-stress emergency unit, which explains the high levels of EE in those working there and corroborates our theory. It follows that low EE is related to the general hospital profile, where few environmental stressors exist and turnover is low 18,21. EE is considered the first stage of BO as well as the main cause of its onset. It leads to anxiety and low energy, which in turn cause declines in health and quality of life 22,23. Even when only low levels exist, it can seriously disrupt social dynamics, in particular regarding the hospital power equilibrium between technicians and nurses but also between coworkers in general 4,5.

High levels of DP were found in specialty nurses (61.1%, *p*=0.024), in accordance with another study in which subjects with higher levels of education were found to be at greater risk of BO 24,25. This could result from the greater responsibilities imposed on those with higher levels of education, the higher professional expectations and possibly the increased status that comes with recognition from other health professionals 23. DP was also higher in physicians with several jobs, possibly due to the fact that commuting between jobs can cause delays, an unnecessary stressor. DP was more prominent in female (50.2%) versus male (32.0%) nursing technicians. It should be noted that prior studies have been controversial regarding differences in DP related to gender 20,25, although two recent studies evaluating risk of BO in health professionals concluded that females experience higher levels of DP than males 27. Women are commonly managing household work in addition to their professional responsibilities, which can increase stress and lead to higher levels of DP in the work environment. Since the majority of nurses are female, this could be a decisive element in explaining these findings 27,28. In women, BO has also been associated with marital status, number of years in practice, sleep deprivation, back pain and negative effects in family life 29,30,31.

Some previous studies 32,33 have found a significant relationship between job type and differences in EE, DP, and PA. In the present study, PA levels were lowest in physicians relative to nurses and nursing technicians. Low PA causes a decline in productivity and can be exacerbated by insufficient social support and opportunities for personal development, number of jobs, and high workload 26,34. Higher PA was associated with postgraduate training. It is possible that the self-confidence and experience provided by postgraduate studies leads to feelings of self-assurance, thus reducing stress and, consequently, levels of EE, DP and BO risk.

In the present study, doctors were found to have low PA. However, a prior study of BO in medical residents found that dissatisfaction with workload, working hours, relationships with co-workers and lack of autonomy are the main causes for high levels of BO 35. Other research has indicated that number of jobs, high workload and lengthy commute all lower PA. In addition, type of work contract, as in the case of outsourced employment, can also lower PA and undervalue employees since no benefits are received 33.

The main findings of this study show that most participants suffered from EE, although at a low level. Because most also exhibited symptoms of DP, they were classified as low risk for BO. However, a high percentage of specialized nurses and female nursing technicians suffered from DP at moderate to high levels, especially individuals who had many jobs. Low levels of PA were also found in those who had only completed undergraduate studies compared to those with higher levels of training. Although these factors translate into only a low to moderate risk for BO, preventive measures are still necessary to avoid the syndrome.

It should be noted that several limitations exist concerning our results. The lack of MBI standardization and the subjectivity of perception could have influenced the evaluation of MBI dimensions and caused heterogeneity among studies. Additional investigations must be conducted to standardize the instrument used to assess BO. This in turn will produce homogeneous results and enable a meta-analysis to be performed, thus improving the quality of evidence.

Moreover, as with all observational cross-sectional studies, because information was collected through self-reported questionnaires applied during working hours and under stressful conditions, ambiguity does exist. Additionally, the lack of specific clinical diagnoses to assess physical and psychological conditions could have influenced the reporting of EE, DP and PA.

Future research on BO should consider factors such as occupational social network (colleagues and superiors), workplace, and environmental conditions in addition to testing interventions to improve wellbeing and thus minimize the risk of BO.

## AUTHOR CONTRIBUTIONS

Paiva LC was responsible for planning the research, collecting the quantitative data, discussing the results, and writing the manuscript. Canário AC was responsible for analyzing the qualitative data and discussing the results. China EL was responsible for analyzing the qualitative data, discussing the results, and writing the manuscript. Gonçalves AK was responsible for planning and supervising the research, analyzing the data, discussing the results, producing the final revisions, and submitting the manuscript.

## Figures and Tables

**Table 1:** Levels and risk of emotional exhaustion (MBI) among healthcare professionals.

	Sociodemographic variables	Emotional exhaustion				*p*-value	OR (95% CI)
		High N (%)	Moderate N (%)	Low N (%)	Overall N (%)		
NURSES	Educational attainment						1.23 (0.45-1.45)
	Bachelors level	05 (25.0)	01 (5.0)	14 (70.0)	20 (19.8)		
	Specialist level	12 (22.2)	13 (24.1)	29 (53.7)	54 (53.5)	0.16^1^	
	Masters level	09 (34.6)	06 (23.1)	11 (42.3)	26 (25.7)		
	PhD level	00 (0.0)	01 (100.0)	00 (0.0)	01 (1.0)		
	Workplace						
	General hospital	21 (29.1)	16 (22.2)	35 (48.6)	72 (72.0)		
	Maternity	04 (14.3)	05 (17.9)	19 (67.9)	28 (28.0)	0.18	
DOCTORS	Educational level						1.25 (0.79-1.99)
	Bachelors level	10 (30.3)	10 (30.3)	13 (39.4)	33 (40.7)		
	Specialist level	10 (24.4)	09 (22.0)	22 (53.7)	41 (50.6)		
	Masters level	01 (25.0)	00 (0.0)	03 (75.0)	04 (4.9)	0.75^1^	
	PhD level	01 (33.3)	01 (33.3)	01 (33.3)	03 (3.7)		
	Workplace						
	General hospital	10 (20.0)	12 (24.0)	28 (56.0)	50 (61.7)	0.12^1^	
	Maternity	12 (38.7)	08 (25.8)	11 (35.5)	31 (38.3)		
NURSE TECHNICIANS	Educational level						1.55 (0.94-2.57)
	Bachelors level	09 (17.6 )	17 (33.3)	25 (49.0)	51 (20.1)		
	Specialist level	11 (20.4)	05 (9.3)	38 (70.4)	54 (21.2)		
	Masters level	00 (0.0)	03 (75.0)	01 (25.0)	04 (1.6)	0.04^1^	
	PhD level	00 (0.0)	00 (0.0)	01 (100.0)	01 (0.4)		
	Nursing school	21 (14.6)	38 (26.4)	85 (59.0)	144 (56.7)		
	Workplace						
	General hospital	27 (13.6)	45 (22.6)	127 (63.8)	199 (78.3)		
	Maternity Unit	14 (25.5)	18 (32.7)	23 (41,8)	55 (21.6)	0.01^1^	

1 Chi-square and Fisher’s exact test MBI: Maslach BO Inventory.

**Table 2:** Levels and risk of depersonalization (MBI) among healthcare professionals.

	Sociodemographic variables	Depersonalization				*p*-value	OR (95% CI)
		High N (%)	Moderate N (%)	Low N (%)	Overall N (%)		
Sex	Female	45 (48.4)	34 (36.6)	14 (15.1)	93 (92.1)	0.73^1^	1.04 (0.58-1.88)
	Male	05 (62.5)	02 (25.0)	01 (12.5)	08 (7.9)		
NURSES	Educational level						
	Bachelors level	06 (30.0)	12 (60.0)	2 (10.0)	20 (19.8)		
	Specialist level	33 (61.1)	14 (25.9)	07 (13.0)	54 (53.5)	0.02^1^	
	Masters level	11 (42.3)	10 (38.5)	05 (19.2)	26 (25.7)		
	PhD level	00 (0.0)	00 (0.0)	01 (100.0)	01 (1.0)		
	Number of jobs						
	01 job	31 (50.8)	22 (36.1)	08 (13.1)	61 (59.8)		
	02 jobs	18 (47.4)	13 (34.2)	07 (18.4)	39 (38.2)	0.91^1^	
	03 jobs	01 (50.0)	01 (50.0)	00 (0.0)	02 (2.0)		
	> 03 jobs	00 (0.0)	00 (0.0)	00 (0.0)	00 (0.0)		
Sex	Female	25 (54.3)	11 (23.9)	10 (21.7)	46 (56.8)		
	Male	16 (45.7)	08 (22.9)	11 (31.4)	35 (43.2)	0.60^1^	1.16 (0.52-1.41)
DOCTORS	Educational level						
	Bachelors level	14 (42.4)	08 (24.2)	11 (33.3)	33 (40.7)		
	Specialist level	24 (58.5)	09 (22.0)	08 (19.5)	41 (50.6)		
	Masters level	01 (25.0)	01 (25.0)	02 (50.0)	04 (4.9)	0.56^1^	
	PhD level	02 (66.7)	01 (33.3)	00 (0.0)	03 (3.7)		
	Number of jobs						
	01 job	12 (60.0)	05 (25.0)	03 (15.0)	20 (24.7)		
	02 jobs	27 (50.9)	14 (26.4)	12 (22.6)	53 (65.4)	0.01^1^	
	03 jobs	02 (25.0)	00 (0.0)	06 (75.0)	08 (9.9)		
Sex	Female	104 (50.2)	70 (33.8)	33 (15.9)	207 (81.5)	0.04^1^	1.25 (0.79-1.99)
	Male	15 (31.9)	19 (40.4)	13 (27.7)	47 (18.5)		
NURSE TECHNICIANS	Educational level						
	Bachelors level	25 (49.0)	16 (31.4)	10 (19.6)	51 (20.1)		
	Specialist level	26 (48.1)	20 (37.0)	08 (14.8)	54 (21.3)	0.78^1^	
	Masters level	01 (25.0)	01 (25.0)	02 (50.0)	04 (1.60)		
	PhD level	01 (100.0)	00 (0.0)	00 (0.0)	01 (0.4)		
	Nursing school	66 (45.8)	52 (36.1)	26 (18.1)	144 (56.7)		
	Number of jobs						
	01 job	79 (49.4)	50 (31.2)	31 (19.4)	160 (63.0)		
	02 jobs	40 (43.5)	38 (41.3)	14 (15.2)	92 (36.2)	0.30^1^	
	03 jobs	00 (0.0)	01 (50.0)	01 (50.0)	02 (0.8)		

1 Chi-square and Fisher’s exact test MBI: Maslach BO Inventory.

**Table 3:** Levels of PA among healthcare professionals.

	Sociodemographic variables	Professional achievement				*p*-Value	OR (95% CI)
		Low N (%)	Moderate N (%)	High N (%)	Overall N (%)		
NURSES	Educational level						2.70 (1.40-5.20)
	Bachelors level	06 (30.0)	12 (60.0)	2 (10.0)	20 (19.8)		
	Specialist level	33 (61.1)	14 (25.9)	07 (13.0)	54 (53.5)	0.02^1^	
	Masters level	05 (19.2)	10 (38.5)	11 (42.3)	26 (25.7)		
	PhD level	00 (0.0)	00 (0.0)	01 (100.0)	01 (1.0)		
DOCTORS	Educational level						1.54 (0.36-1.16)
	Bachelors level	22 (66.7)	07 (21.2)	04 (12.1)	33 (40.7)		
	Specialist level	23 (56.1)	07 (17.1)	11 (26.8)	41 (50.6)		
	Masters level	01 (25.0)	00 (0.0)	03 (75.0)	04 (4.9)	0.03^1^	
	PhD level	01 (33.3)	02 (66.7)	00 (0.0)	03 (3.7)		
	Nursing school	00 (0.0)	00 (0.0)	00 (0.0)	00 (0.0)		
NURSE TECHNICIANS	Educational level						1.74 (1.09-2.79)
	Bachelors level	23 (45.1)	09 (17.6)	19 (37.3))	51 (20.1)		
	Specialist level	22 (40.7)	12 (22.2)	20 (37.0)	54 (21.30)		
	Masters level	01 (25.0)	01 (25.0)	02 (50.0)	04 (1.6)	0.04^1^	
	PhD level	00 (0.0)	00 (0.0)	01 (100.0)	01 (0.4)		
	Nursing school	63 (43.8)	36 (25.0)	45 (31.2)	144 (56.7)		

5% significance level;

1 Chi-square and Fisher’s exact test.
